# Surgical Approach in the Treatment of Calciphylaxis: A Case Report

**DOI:** 10.3390/healthcare13172175

**Published:** 2025-08-31

**Authors:** Tomáš Demčák, Radovan Čeľovský, Ján Babík, Peter Lengyel, Lenka Krešáková, Katarína Vdoviaková, Nikola Hudáková, Filip Humeník

**Affiliations:** 1 Clinic of Burns and Reconstructive Medicine, AGEL Hospital, 040 15 Košice-Šaca, Slovakia; 2 Department of Morphological Sciences, University of Veterinary Medicine and Pharmacy in Košice, 041 81 Košice, Slovakia

**Keywords:** calciphylaxis, wound, reconstructive surgery

## Abstract

**Background:** Calciphylaxis (calcific uremic arteriolopathy), is a rare disease characterized by subcutaneous vascular thrombosis and necrotic skin lesions, which mainly affects patients with kidney disease. This condition often has a poor prognosis, unclear pathophysiology, and lacks standardized treatment. **Case Description:** We present a case of calciphylaxis in a 53-year-old female patient who reported gradually worsening unbearable pain in her lower limbs and thighs, persisting for approximately 18 months. After appropriate examinations and biopsy of non-healing wounds, histopathology confirmed the diagnosis of calciphylaxis. The wounds were treated with dermo-epidermal (DE) grafts. Followingly, the patient underwent treatment in a hyperbaric chamber, after which the wounds decreased in size. **Conclusions:** Early diagnosis and a comprehensive approach to therapy are necessary to improve the management of calcification, a rare disease, and complications such as non-healing wounds.

## 1. Introduction

Calciphylaxis is a rare but very serious disease characterized by calcium deposition in arterioles in the skin and subcutaneous fat, fibrosis of the tunica intima, and thrombosis of subcutaneous vessels. The exact cause of this disease is not yet known. It predominantly occurs in patients with end-stage renal disease (ESRD) and can also rarely occur in patients with acute or chronic renal impairment, but also in patients with normal renal function [1,2]. It is also known as calcifying uremic arteriolopathy (CUA). CUA is characterized by the presence of very painful skin lesions due to calcification of cutaneous arterioles, which subsequently leads to tissue ischemia and the formation of deep ulcerations. Painful nodules are often palpable on the skin. This disease is accompanied by significant mortality due to the presence of severe pain, non-healing wounds, and frequent hospitalizations. The annual mortality rate of this disease is reported to be greater than 50%, most commonly due to septic complications of non-healing wounds [3].

Risk factors associated with the occurrence of this rare disease are Caucasian ethnicity, female gender, kidney disease, obesity, diabetes mellitus, hypoalbuminemia, autoimmune diseases (lupus erythematosus, rheumatoid arthritis, antiphospholipid syndrome), liver disease, malignancy, and hemodialysis. Drug-related risk factors include severe use of warfarin, corticosteroids, iron substrates, and activated vitamin D. Also included are abnormalities associated with chronic kidney disease and bone metabolism disorders such as hyperphosphatemia, hypercalcemia, and hyperparathyroidism [4,5]. Bajaj et al., in their retrospective study, examined patients who had a clinical picture of calciphylaxis but had preserved normal kidney function. He found that factors contributing to the development of this disease in people with preserved renal function were white race in 89% of enrolled patients, female gender in 78% of enrolled patients, treatment with vitamin K antagonists, and obesity [6].

According to the epidemiological data, calciphylaxis is a very rare disease. In patients requiring dialysis, it occurs in approximately 35 patients out of 10,000 in the USA. [7] In Europe, the incidence is even lower, with the literature describing the occurrence of calciphylaxis in only 4 patients per 100,000 population [8,9].

At the Burn and Reconstructive Surgery Clinic of the Agel Košice-Šaca Hospital, we are involved in the daily treatment not only of burned patients but also of chronic wounds of various etiologies. Recently, we had the opportunity to encounter a case of this rare disease, in which the patient developed deep ulcerations in various places. This significantly impairs the patient’s quality of life due to severe pain and puts them at risk of developing wound infection and even sepsis. In this clinical case report, we describe a patient admitted to the clinic for treatment of non-healing wounds that were treated as suspected vasculitis. Within the case report, we present a chronological overview of the events in the patient’s treatment and diagnosis, with the aim of increasing awareness about calciphylaxis and bringing further insight in the context of more effective diagnostics and management of this rare disease.

## 2. Case Report

A 53-year-old patient was presented to the dermatovenerology department of our hospital for differential diagnosis of suspected vasculitis in February 2024. The patient complained of intolerable pain in the lower legs and thighs lasting for a period of approximately 18 months and progressively worsening over time. Locally, bruises were described on the thighs, which merged into larger deposits, from which wounds were subsequently formed, without prior traumatization. Locally, the wounds were treated with medical-grade Manuka honey and viscose net dressing coated with 99% Manuka honey and 1% Manuka oil. Due to the painful conditions, the patient was taking excessive amounts of non-steroidal anti-inflammatory drugs (NSAIDs). The patient was being treated for hypertension, glaucoma, and macrocytic anemia. The patient did not have any stage of renal failure. Further medical history included a history of nephrolithiasis of the left kidney, a hypodense nodule in the right lobe of the thyroid gland, and chronic venous insufficiency. Allergic history was negative. Biopsy of the lesions was recommended by the dermatologist because of suspicion of necrotizing vasculitis.

**Figure 1:**
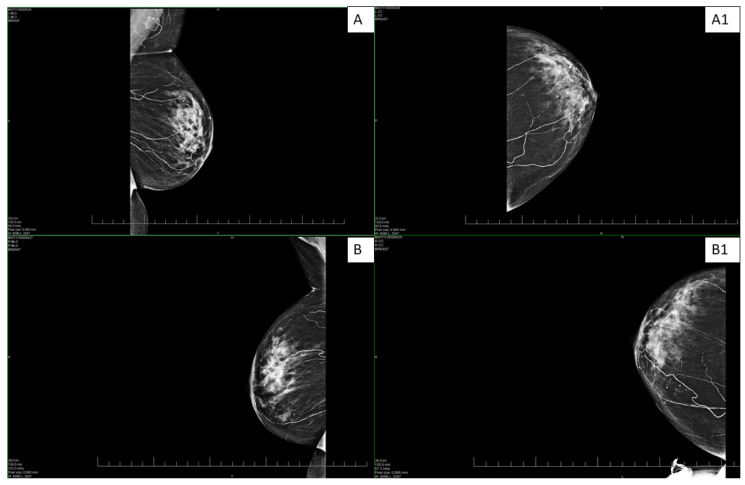
Bilateral digital mammography—standard craniocaudal (**A**,**B**) and mediolateral oblique projections (**A1**,**B1**) on both sides. The images show scattered microcalcifications and extensive mediocalcinosis on both sides, more pronounced in the left breast.

An abdominal ultrasound examination in March 2024 revealed hepatosplenomegaly. A subsequent vascular examination ruled out deep vein thrombosis. Routine CT oncological examination and mammography were negative (Figure 1). The patient underwent a gastroenterological examination, with laboratory parameters showing an increase in GMT to 605.88 U/L and hemoglobin of 86 g/L. The conclusion of the examination described gastritis in the upper gastrointestinal tract and NSAID gastropathy due to analgesic abuse; other parameters and local findings were normal. A biopsy of the lesion in the same month showed that the patient had coagulase-negative Staphylococcus in the wound. The biopsy results also described small vessels with thickened walls and perivascular inflammatory infiltrate. The finding was concluded as nonspecific, not consistent with the picture for vasculitis. On 20 March 2024, the patient was hospitalized at the dermatovenerology clinic for further differential diagnosis of her condition. Laboratory findings again showed an increase in GMT 1319.74 U/L, ALP 334.13 U/L, urea 10 mmol/L, and creatinine 121 μmol/L. The proposed treatment consisted of high doses of methylprednisolone (500 mg intravenously for 3 consecutive days) followed by a reduced dose of 250 mg intravenously and then a gradual transition to tablet form.

An angiological examination in April 2024 revealed deep vein thrombosis of the right lower limb in the area of the right popliteal vein. Low molecular weight heparin therapy was added to the treatment for 30 days. A subsequent MRI of the abdomen (May 2024) again showed hepatosplenomegaly. Laboratory findings showed elevated GMT 2159.57 U/L and ALP 167.97 U/L. A dermatovenerologist indicated surgical intervention, necrectomy of the non-healing wounds. Due to the presence of hepatopathy, it was not possible to introduce immunosuppressive treatment by a rheumatologist. The patient was indicated for a PET/CT scan to rule out hypermetabolic foci. The examination (June 2024) described a loss of metabolic activity in the area of defects on the front of the thighs and on the medial side of the right lower limb, deeper subcutaneous tissue, and muscle structures with adequate metabolic activity.

**Figure 2:**
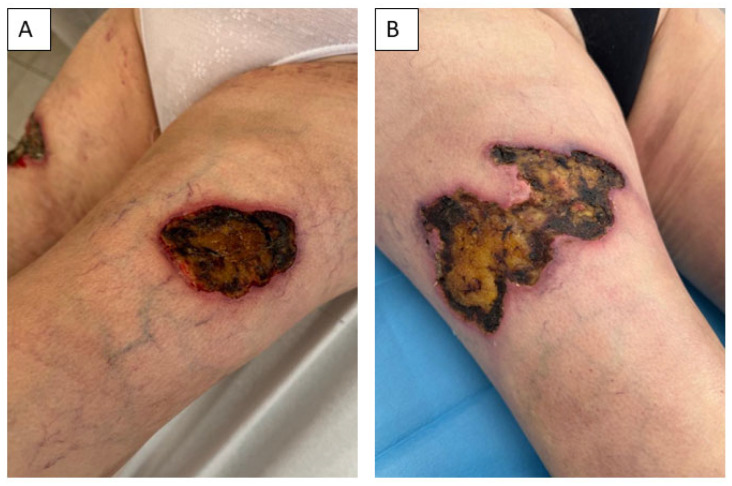
The chronic non-healing wounds were localized on the left (**A**) and right (**B**) thighs of the patient before the initiation of wound treatment.

In 18 June 2024, the patient was admitted for the first time to the Clinic of Burn and Reconstructive Surgery in Košice-Šaca, where treatment with dressings for non-healing wounds was initiated (Figure 2), and high-dose methylprednisolone therapy was initiated. This was subsequently tapered and converted to an oral regimen of 20 mg methylprednisolone once daily in the morning, which she continued without interruption until July 2024, when the diagnosis of calciphylaxis was established at our clinic. Importantly, the patient was not receiving any additional medication known to be associated with calciphylaxis, nor any drugs that could have affected thyroid function. Table 1 summarizes the relevant laboratory parameters at the time of hospitalization. No significant changes associated with calciphylaxis were present; only GGT was elevated significantly, which we attributed to NSAID-associated hepatopathy. The patient had a slightly lower renal function according to GFR-EPI at the time of admission. A multi-resistant strain of Enterobacter cloacae was found in the patient’s wound.

**Figure 3:**
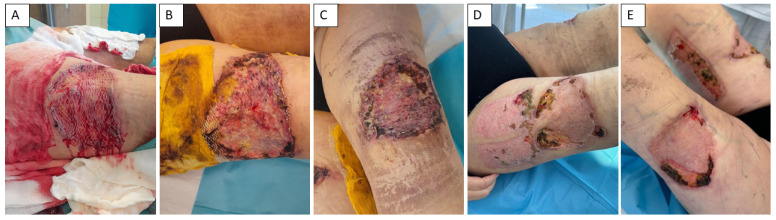
Management of calciphylaxis-related skin defects. Application of DE grafts and complicated healing (**A**,**B**). Status of wound healing process before admission (**C**). Complicated healing process of side location of harvested autologous DE grafts (**D**). Defect 2 months after surgical procedures (**E**).

In July 2024, a necrectomy was performed on all defects on the ventral side of the right and left thighs and on the medial side of the right lower limb, covering a total of 3% of the total body surface area. The tissue sample was subjected to histological analysis. Subsequently, we proceeded to reconstruct the wounds using dermo-epidermal split- thickness skin grafts (DE), which were taken from the right thigh, meshed in a 3:1 ratio, and applied to the wounds (Figure 3). As a secondary dressing, we used synthetic antimicrobial dressings (Mepilex Ag, Mölnlycke, Gothenburg, Sweden). In our case, debridement and skin grafting were performed as separate procedures. Negative pressure wound therapy (NPWT) was not employed in this patient with calciphylaxis due to several limiting factors. The patient experienced severe pain and psychological distress related to previous dressing changes, which rendered NPWT poorly tolerable. In addition, given the anatomical location of the ulcer, it was not feasible to ensure adequate application and maintenance of the foam dressing without significantly restricting the patient’s mobility. For these reasons, NPWT was considered unsuitable in this case.

**Figure 4:**
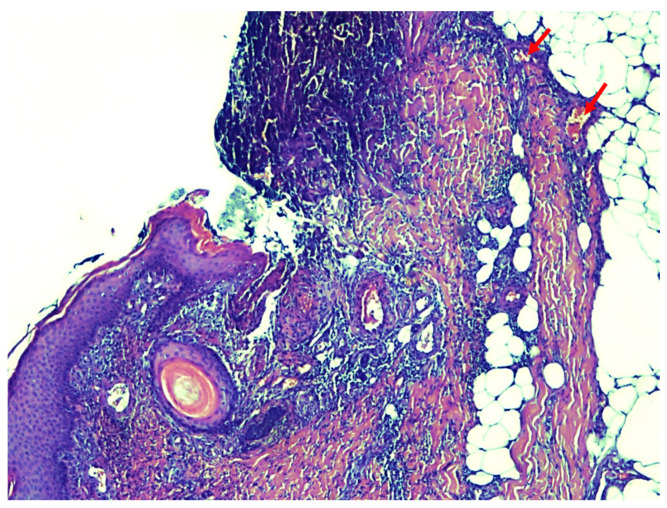
The results of the histological examination show the presence of extensive necrosis of the epidermis and the presence of calcium deposits in the small deep dermal vessels (pointed to by red arrows).

The histological examination results (Figure 4) described the presence of extensive superficial necrosis of the epidermis with complete destruction of the epithelium and spread of inflammatory infiltrate into the subcutaneous tissue in all samples. In various locations, mainly in the deeper dermis down to the subcutaneous adipose tissue, calcifications with proliferation of intimal fibroblasts were found in the walls of smaller vessels, and in some vessels, the occurrence of thrombosis was found. The patient was therefore diagnosed with calciphylaxis (July 2024). The postoperative period was complicated by partial lysis of the DE grafts, after which the patient underwent 15 sessions of hyperbaric oxygen therapy, lasting 120 min. This resulted in significant progress in the healing of the DE graft wounds. Hyperbaric oxygen therapy was delivered in a 13-seat Haux chamber. During each session, at a pressure of 1.6 bar, the patient inhaled 100% oxygen via a face mask. After discharge from the department, the patient was prescribed sodium thiosulfate. The patient continues to undergo wound dressing therapy, and the wounds are significantly smaller than the condition at the time of discharge.

## 3. Discussion

Calciphylaxis (calcifying uremic arteriolopathy) typically manifests as very painful skin lesions or painful subcutaneous nodules without skin changes, which can progress to skin ulcers. Skin lesions are often purple or red in the early stages and may resemble livedo reticularis. As the disease progresses, these skin changes may turn into pale yellow ulcers, or dry black necrosis may occur. The most commonly affected areas are those with a larger amount of subcutaneous fat (e.g., thighs, back of the calves, abdominal wall, breasts). Gangrenous changes and, in rare cases, autoamputation may be observed in the distal parts of the limbs, such as the fingers or penis. At every stage of the disease, skin lesions are extremely painful. Although skin manifestations are the main symptom of this disease, calcinosis is a systemic disease that can also affect other organs, including the eyes, musculoskeletal system, brain, intestines, and lungs [1].

The pathophysiology of calcification is not entirely clear. It is a multifactorial disease involving several factors. The development of this disease involves calcification of the tunica media of arterioles, fibrosis of the intima, and subsequent thrombosis due to progressive calcification and endothelial dysfunction. Calcification of small arteries and arterioles contains crystals of pure apatite (calcium phosphate), which are deposited in a circular pattern, mainly in the intima and interstitium, unlike arteriosclerosis, where interstitial deposits are absent [10,11]. In the affected area in the afferent arteries, medial calcification is often described on X-ray images [5].

Studies show that the main cause is a defect in calcium, phosphorus, and vitamin D metabolism. Similar results were observed in our case, where the level of vitamin D concentration in blood was 21 nmol/L (physiological range 75–200 nmol/L) and parathormone was 9.5 ng/L (physiological range 15–65 ng/L). Calcium and phosphorus levels were in the physiological range. However, calcification can also develop in cases where parathyroid hormone, phosphorus, and calcium levels are normal. A deficiency of vascular calcification inhibitors such as fetuin 1, osteoprotegerin, and matrix GLA protein may play a role in the development of calcification. Fetuin A is a glycoprotein that binds calcium and phosphorus and may help prevent calcification of blood vessels and soft tissues. Fetuin 1 is reduced in dialysis patients. Matrix GLA protein may also prevent vascular calcification, and its activity depends on vitamin K-dependent carboxylation. The use of warfarin is considered a risk factor for calcification, which may be related to its interference with vitamin K and activation of matrix GLA protein [3]. Based on pathophysiology, the following forms can be distinguished: (A) Uremic calcification: the most common and typically occurring in dialysis patients with end-stage renal failure. (B) Non-uremic calcification: much less common, seen in patients with less advanced kidney damage and occasionally in patients without kidney damage [12]. Our patient did not suffer from renal failure. In the patient’s history, only nephrolithiasis was observed. Therefore, it was a case of non-uremic calciphylaxis.

The definitive diagnosis of calciphylaxis is established on the basis of a skin biopsy of one of the present lesions. Histological examination describes calcification of the tunica media of dermal arterioles and small arteries and may describe fibrointimal hyperplasia, the presence of microthrombi, narrowed lumens, or obliteration of arteries, often with the presence of necrosis. However, the necessity of biopsy in this disease is debatable. Biopsy in calciphylaxis serves to differentiate the disease in the early stages from other skin diseases, but the procedure itself carries risks that must be taken into account, especially in patients in whom the disease manifests itself in the form of subcutaneous nodules. The procedure itself may trigger the formation of ulcerations, the wound may not heal, and bleeding and infection may occur [3,5].

In terms of laboratory tests, it is important to examine renal parameters, namely urea and creatinine, calcium, vitamin D, parathyroid hormone (PTH), liver function, inflammatory parameters, and coagulation parameters. It is important to note that serum calcium and phosphorus levels are not decisive for diagnosis. An important parameter is bone mass renewal, which is affected by parathyroid gland activity [5]. A predisposition to calcification is very high or very low bone renewal. High bone metabolism leads to the rapid release of calcium and phosphorus from the bones, which are subsequently deposited in the blood vessels. With low bone turnover, the ability of bones to deposit minerals is reduced, and these minerals are again deposited outside the bones, leading to mediocalcinosis. PTH is used as a guide to determine bone turnover; nevertheless, clinical practice remains a key aspect in establishing a diagnosis.

Mediocalcinosis is often observed on X-rays around the arteries in the affected area with extension into the surrounding tissue. However, PET/CT scans offer high-resolution images that help define the extent of vascular involvement, which is important for prognosis and treatment planning [13,14]. Mammography, on the other hand, offers high resolution and contrast for the detection of calcifications, which was the case with our patient (Figure 1). Given this, the imaging techniques can contribute to the early identification of calcifications [5,14].

There is still no “evidence-based” treatment for calciphylaxis. Regarding clinical practice treatment, sodium thiosulfate is used for the treatment of this disease [15,16]. In a study that evaluated its effectiveness in the treatment of calciphylaxis, it was proven that its use in more than 70% of patients improved or resolved the existing skin lesions, and a reduced mortality rate was also recorded. Sodium thiosulfate is standardly administered intravenously during hemodialysis, but administration per os and locally to skin lesions has been described. Sodium thiosulfate is effective in the treatment of calciphylaxis even in patients with healthy kidneys; it is administered intravenously. It is administered long-term for several months, until the skin lesions heal. It is reported that after 2 weeks of use, it also has an analgesic effect. This drug is not included in the list of registered drugs in Slovakia [5].

In addition to administering sodium thiosulfate, when diagnosing calciphylaxis, it is important to eliminate all risk factors causing the disease. It is necessary to diagnose and correct calcium and phosphorus metabolism, diagnose and radically resolve hyperparathyroidism, and immediately discontinue all medications associated with the risk of developing calciphylaxis—warfarin, vitamin D, and corticosteroids. The monitoring of dialysis therapy is necessary. Proper care of skin ulcerations, regular wound debridement, and dressing therapy to prevent wound infection cannot be neglected. In this case, therapy was based on wound debridement connected with DE-skin grafting. In patients with calciphylaxis, surgical wound management is particularly challenging due to compromised tissue perfusion, ongoing vascular calcification, and a high risk of infection. While one-step procedures combining debridement and immediate skin grafting can shorten treatment time and reduce the number of operations, their success depends on the presence of a well-vascularized wound bed. In calciphylaxis, however, the wound bed is frequently suboptimal at the time of debridement, leading to a high likelihood of graft failure [17].

Delayed grafting after initial debridement and temporary coverage (at our department, e.g., with silver-containing dressings, xenografts, NPWT) allows for additional wound bed preparation, control of local infection and inflammation, and better assessment of tissue viability [17,18]. For this reason, we decided on delayed grafting in such cases. The primary rationale for selecting split-thickness skin grafts over alternative reconstructive options was the favorable balance between feasibility and safety. First, donor site availability was a decisive factor, as split-thickness grafts allow harvesting sufficient skin even in elderly and comorbid patients while minimizing donor site morbidity. Second, the advanced age of the patient made more extensive reconstructive procedures, such as flaps, less desirable due to the increased operative risk and prolonged anesthesia time. Third, in the setting of calciphylaxis, vascular compromise markedly increases the risk of flap necrosis and failure, further supporting grafting as the most reliable option. Taken together, these considerations made split-thickness dermo-epidermal grafting the most appropriate reconstructive strategy, offering dependable wound closure with lower risk compared to flap-based procedures. Pain management is key. Prophylactic administration of antibiotics is not recommended. There are studies that describe the beneficial effect of hyperbaric oxygen therapy (HBO) on the treatment of wounds [19], which was also used in the case of our patient. HBO has been proposed as an adjunctive treatment modality in calciphylaxis. The rationale is based on the pathophysiology of the disease: extensive vascular calcification, thrombosis, and tissue ischemia result in profound hypoxia and impaired wound healing. HBO increases the partial pressure of oxygen in blood, thereby enhancing tissue oxygenation even in areas with compromised microcirculation. Improved oxygen delivery can support fibroblast activity, angiogenesis, and collagen synthesis, all of which are critical for wound repair. Biglione et al. reported that HBO was associated with longer survival time (*p* = 0.016) [19]. An et al. stated that more than half of patients who had full HBO treatment had higher survival rates [20]. The prognosis of this disease remains very unfavorable, with an annual mortality rate ranging from 45% to 80%. Patients with ulcerations are at high risk of infectious complications, which can lead to sepsis and even death [3]. All these facts only emphasize the necessity of research addressing this issue in order to improve early diagnosis and effective management involving a multidisciplinary approach to this rare disease.

## 4. Conclusions

Calciphylaxis is a very rare disease with a very poor prognosis for the patient, the nature of which is still not fully understood. Given the very painful lesions that form practically anywhere on the body, early diagnosis of the disease, proper pain management, a suitably designed treatment strategy, and definitive treatment of these wounds with subsequent long-term nursing care are necessary from a surgical point of view. Calciphylaxis is largely unknown outside dermatology, nephrology, rheumatology, and vascular surgery. Therefore, interdisciplinary communication and exchange of experiences are very important.

Patient’s perspective: After successful management of the surgical approach to wounds connected to non-uremic calciphylaxis, the patient was released to home-based care; the following treatment was under the surveillance of a general practitioner.

## Figures and Tables

**Table 1:** Admission laboratory parameters. The “*” sign is indicated for those parameters for which a deviation from the physiological range was recorded.

Parameter	Value	Physiological Range
WBC	7.4 × 10^9^/L	4–11 × 10^9^/L
Neu	5.5 × 10^9^/L	2.5–7 × 10^9^/L
Lym	1.4 × 10^9^/L	1–4.8 × 10^9^/L
Mono	0.4 × 10^9^/L	0.2–0.8 × 10^9^/L
Eo	0.1 × 10^9^/L	≤0.45 × 10^9^/L
Baso	0.0 × 10^9^/L	≤0.3 × 10^9^/L
RBC *	2.35 × 10^12^/L	4.2–6.1 × 10^9^/L
Hgb *	77 g/L	120–180 g/L
Hct *	0.238	0.36–0.54
Plt	165 × 10^9^/L	150–450 × 10^9^/L
Creatinine	80 umol/L	44–115 μmol/L
Urea	4.5 mmol/L	3.0–10.0 mmol/L
GFR-EPI *	1.24 mL/s	1.5–2 mL/s
GGT*	21.72 μkat/L (1302.94 U/L)	6–50 U/L
AST	0.43 μkat/L (25.9 U/L)	8–33 U/L
ALT	0.31 μkat/L (18.62 U/L)	7–55 U/L
Albumin	36 g/L	35–55 g/L
Calcium	2.43 mmol/L	2.12–2.62 mmol/L
Calcium ionized	1.23 mmol/L	1.1–1.4 mmol/L
Phosphorus	1.1 mmol/L	0.8–1.5 mmol/L
Parathormone (PTH) *	9.5 ng/L	10–65 ng/L
Folic acid *	3.7 nmol/L	9–45 nmol/L
Vitamin D *	21 nmol/L	50–125 nmol/L
C-reactive protein (CRP) *	42.3 mg/L	≤3 mg/L
INR	0.98	0.8–1.1
AT III *	133%	80–120%
Fibrinogen	3.3 g/L	0.2–4.0 g/L
Thrombine time	16.8 s	9–18 s
Prothrombin time	12.3 s	11–13.5 s

## Data Availability

The data presented in this study are available upon request from the corresponding author.
